# Characterisation of the Mycobiota on the Shell Surface of Table Eggs Acquired from Different Egg-Laying Hen Breeding Systems

**DOI:** 10.3390/toxins10070293

**Published:** 2018-07-16

**Authors:** Łukasz Tomczyk, Łukasz Stępień, Monika Urbaniak, Tomasz Szablewski, Renata Cegielska-Radziejewska, Kinga Stuper-Szablewska

**Affiliations:** Department of Food Safety and Quality Management, Poznan University of Life Sciences, 60-624 Poznan, Poland; lste@igr.poznan.pl (Ł.S.); murb@igr.poznan.pl (M.U.); tomasz.szablewski@up.poznan.pl (T.S.); renatara@au.poznan.pl (R.C.-R.); kinga.stuper@up.poznan.pl (K.S.-S.)

**Keywords:** eggs, hen breeding systems, microscopic fungi, mycotoxins

## Abstract

Microbial safety is an important factor contributing to the egg quality. During egg acquisition, there is significant risk of contamination of the eggshell surface with microscopic fungi. Mycelial hyphae may grow on the eggshell surface and penetrate into the egg content. However, there is no information on the populations of microscopic fungi on the eggshell surface and, consequently, on possible production of mycotoxins. Therefore, the aim of the study was to identify the species of microscopic fungi present on the eggshell surface acquired from different breeding systems and to measure the number of selected mycotoxins. The qualitative analysis resulted in the identification of 41 isolates on the surface of eggs. There were 7 isolates from the organic production system, 11 from the free-range production system, 14 from the deep litter indoor housing system and 9 from the cage farming production system. The research proved that the diversification in the population of mycobiota on the eggshells depended on the egg-laying hen breeding system. The microscopic fungi isolated from the eggshells included toxigenic and pathogenic species such as *Fusarium culmorum* and *F. equiseti*. As the egg storage time increased, fungi, including the pathogenic species, penetrated through the eggshells. In consequence, mycotoxins were identified in the egg whites. Type-A and type-B trichothecenes were found in the eggshell samples containing *F. culmorum*.

## 1. Introduction

Eggs are important food all over the world. Following China, the European Union is the second largest producer of eggs for consumption—its share in the global production amounts to about 10% [1]. Due to the high consumption of eggs it is important to guarantee high standards of consumer safety during production process. Therefore, scientists are conducting research to identify potential risks to consumers’ health. Most of their attention is concentrated on the bacterial microflora [2,3,4]. However, latest studies point out a threat caused by the eggshell contamination with microscopic fungi [5]. So far, the problem of mycobiota has been studied on hatching eggs. The conditions of incubation (high air humidity and diffusion of vapour from egg content) favour the development of microscopic fungi on eggshells. In consequence, embryos become contaminated with mycelium and they die [6,7]. Due to these risks, also table eggs were subjected to investigations. It was already proven that the shells of table eggs become contaminated with fungi and that mycelial hyphae penetrate through eggshell membranes [8].

Eggshell contamination may be initiated due to the conditions of egg acquisition, that is, the quality of straw and feed, the presence of dust, temperature and moisture. We can suppose that the eggs produced in the deep litter indoor housing, free-range or even organic systems are at the highest risk of contamination with microscopic fungi. In the deep litter indoor housing system, the contamination is usually caused by moist litter or dirty nests. Researchers studying different types of litter found that it contained fungi from *Aspergillus*, *Cladosporium*, *Drechslera*, *Penicillium*, *Stemphylium* and *Fusarium* genera [9,10,11]. Straw chaff was found to be the most contaminated as its CFU/g was the highest (28.49 log CFU/g). It also displayed the highest concentration of ergosterol (ERG) (605.74 mg/kg), which is a chemical indicator of material contamination with microscopic fungi [11]. Fungi not only degrade the substrate on which they occur but they also cause numerous diseases due to the presence of spores in the air [12].

Mycotoxins, which are secondary metabolites produced by some fungal strains, are the greatest risk caused by microscopic fungi, particularly in feeds used for breeding farm animals [13]. The content of mycotoxins in more than a half of the farm animal feeds samples collected in 2010 exceeded the maximum legal limit. In Europe, the feed samples usually contained deoxynivalenol (DON) and T-2 toxin, which belong to the trichothecenes, as well as zearalenone (ZON). The feed samples from Asia and the Pacific region were usually contaminated with DON, ZON, fumonisins and aflatoxins [14]. Mycotoxins from the trichothecene group, fumonisins and zearalenone belong to the most toxic metabolites produced by the *Fusarium* species and were proven to be the most harmful when consumed [15]. Due to the increasing number of reports on the contamination of food products and ingredients with microscopic fungi, it seems interesting to investigate their populations on eggshells and their potential to produce mycotoxins.

The aim of the study was to conduct molecular identification of the species found in mycobiota present on the shell surface of table eggs acquired from different egg-laying hen breeding systems and to identify and quantify the mycotoxins in the respective egg white samples.

## 2. Results and Discussion

### 2.1. Mycobiota Species Identification

Eggs selected for analyses were stored at programmed humidity and temperature, which were similar to the conditions in warehouses and shops. The analyses were conducted on eggs laid by hens kept in 4 systems and in 2 experimental variants, that is, with and without sterilisation (control variant). Surface sterilisation effectively eliminates microorganisms adhering to the eggshell surface. The procedure is applied to acquire isolates from eggshell pores. The qualitative analysis resulted in the identification of 41 isolates on the surface of eggs. In total, there were 7 isolates from the organic production system, 11 from the free-range production system, 14 from the deep litter indoor housing system and 9 from the cage farming production system.

**Figure 1:**
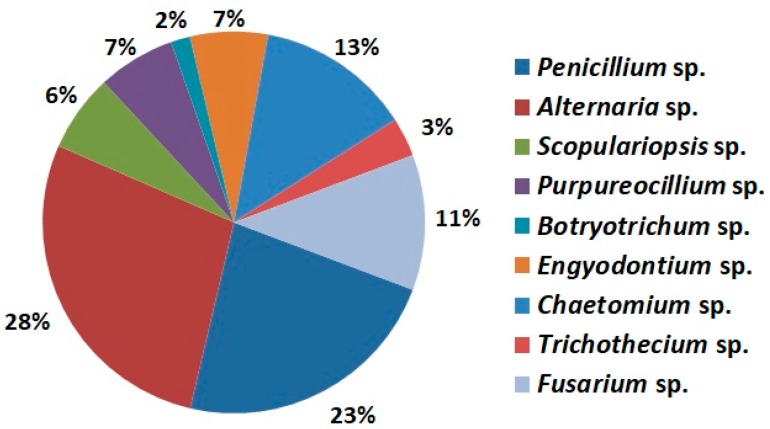
The overall occurrence frequencies of 9 mycobiota (represented by 41 isolates) identified in eggs laid by hens kept in different housing systems.

Fungi belonging to the *Alternaria*, *Penicillium* and *Chaetomium* genera were the most frequently isolated from the eggshell surface (Figure 1). *Alternaria* species isolated in the experiment chiefly colonise crops, including cereal grains [16]. These fungi have the potential to generate toxins such as alternariol and tenuazonic acid. It was found that the *Penicillium* sp. isolated in the experiment accelerated the egg rotting process and caused qualitative changes in the egg content (results not shown). *Chaetomium* sp., which was isolated from the eggshells, is a fungal species commonly found in soil, straw, cereal grains and on wet surfaces of buildings all over the World [17,18,19].

### 2.2. Mycobiota Strains Isolated from Eggs Acquired from Different Egg-Laying Hen Breeding System

This research showed that composition of fungal species isolated from the eggshells differed according to the egg-laying hen housing system (Table 1). There were qualitative and quantitative differences in the fungal species between the sterilised eggs and the control samples (without sterilisation). The highest species diversity was noted in the population of fungi isolated from eggs laid by hens kept in the deep litter indoor housing system. Samples originating from this system mostly contained fungi of the *Alternaria* genus (5 species). There were also two fungal species of the *Fusarium* genus isolated. So far, no reports became available on the occurrence of these species on the eggshell surface. The hygienisation procedure enabled the isolation of *Scopulariopsis brevicaulis*, *Trichothecium roseum* and *Acrostalagmus luteoalbus* species from the shell surface of eggs laid by hens kept in the deep litter indoor housing system. These species were not observed on the shell surface of eggs laid by hens kept in the other housing systems. The diversity of potentially pathogenic fungal species on the eggshell surface is related to the unique microclimate inside the henhouse with the deep litter system, that is, high air humidity and temperature, poor ventilation, exogenous contamination, (litter, feed) and endogenous contamination (dust) [5].

There was similar diversity of fungal species observed in the eggs laid by hens kept in the free-range system. Similarly, to the eggs from the deep litter system, there were also pathogenic species of *Alternaria* and *Fusarium* isolated on the eggshell surface (Table 1). The possibility to control hen breeding conditions in the free-range system is limited. As hens have a free access to full-value feed or green forage, litter and the range, there is higher risk of contact with pathogenic organisms of different origin [20].

There were ten fungal species observed on the shell surface of eggs laid by hens kept in the organic system with no pathogenic species of the *Fusarium* genus among them (Table 1). The hygienisation procedure enabled the isolation of a large population of fungal species in this system. Most of the species were characteristic for the henhouse environment. In the organic system, a limited number of hens is kept per square meter. Consequently, effective control of the henhouse climate is possible. Adequate humidity and temperature reduce the risk of the extensive growth of pathogenic fungi.

There were eight fungal species isolated on the shell surface of eggs laid by hens kept in the cage system. Only two of them had the potential to produce mycotoxins (Table 1). The sterilisation procedure allowed for the isolation of the *Engodontium album* species on the eggshell surface. This species was also isolated from the eggs with the mycoflora native to the deep litter system. Although *E. album* has not been reported to be mycotoxigenic, its spores and mycelium can cause infections in humans causing brain abscess and keratitis [21]. In the present study, the conditions in a henhouse with the cage system resulted in low fungal diversity on the eggshell surface. The design of furnished cages allowed for the natural preferences of hens and the safety of eggs produced [22,23]. As far as the microbial safety of eggs is concerned, the advantage of the cage system results from the fact that it does not use straw, which is a potential vector of pathogenic fungal species from the field.

In conclusion, the qualitative analysis of the fungi isolated showed that the shell of table eggs was a potential substrate for the growth of numerous fungi, including pathogenic and toxin-producing species, for example, *Fusarium* and *Alternaria*. Moreover, the diversity of the fungal population differed according to the egg-laying hen housing system. The fungal species present on the eggshell surface may occur in the environment of the henhouse [9].

### 2.3. Toxin Content Analysis

The presence of *Fusarium* spp. fungi on the eggshell surface involves the potential risk of their presence and production of mycotoxins in the egg content. The content of type-A and type-B trichothecenes was measured in order to check the potential contamination of eggs with mycotoxins (Table 2 and Table 3). Only the egg white was analysed for the content of mycotoxins. Szablewski et al. [8] proved that there were no fungi in the yolk after two weeks of storage. There were no *Fusarium* mycotoxins found in freshly laid eggs. This means that no *Fusarium* mycotoxins were transmitted from the feed through the alimentary tract into the eggs. There were type-A and type-B trichothecenes in the content of the eggs whose shell samples contained *F. culmorum*. This species chiefly synthesizes DON, 3-AcDON and NIV [15,24]. The content of type-A trichothecenes may indicate the presence of species such as *F. sporotrichioides* [25,26]. We did not identify any *Fusarium* species producing type-A trichothecenes in our study. It may have been caused by the fact that only viable fungal species growing on the eggshell surface were analysed. After the period of egg storage, the pathogenic species of microscopic fungi may have penetrated into the egg content. They may have died because of shortage of nutrients or competitive microflora on the eggshell surface.

The opposite situation was observed in some samples. They contained the viable mycelia of microscopic fungi which potentially produce mycotoxins but no mycotoxins were found in the samples. This finding is not surprising, as the biosynthesis of these metabolites may be modified by the occurrence of abiotic or biotic stress factors. For example, under specific conditions the *F. equseti* species is capable of producing selected trichothecene mycotoxins and zearalenone [25,27,28]. Unfortunately, no mycotoxins produced by *F. equseti* were analysed in our study.

### 2.4. Toxin Content Analysis in Eggs Acquired from Different Egg-Laying Hen Breeding System

Our study did not prove any correlation between the content of mycotoxins in egg white and the egg-laying hen breeding system (Table 4). The content of mycotoxins in egg white from hens bred in the organic and cage breeding systems was below the detectable level. There were no significant differences in the concentrations of mycotoxins in egg white from hens bred in the deep litter and free-range systems. Until recently, the issue of the egg content contamination with mycotoxins produced by pathogenic species growing on the egg surface and in the egg content has not been scientifically investigated. So far, the experiments were focused on the transmission of mycotoxins from feed through the alimentary tract into the egg content [29,30,31,32]. In the present study, we have shown that the level of mycotoxins in eggs is not harmful to the human body. However, attention should be paid to the possibility of the bioaccumulation of mycotoxins in the human body [33]. Our study reports the new potential source of chemical and microbial risks in the production of table eggs.

## 3. Conclusions

The research showed that the diversity of the mycobiota population on the eggshell surface depended on the conditions of maintenance of egg-laying hens. The microscopic fungi isolated from the eggshells included toxigenic and pathogenic species such as *F. culmorum* and *F. equiseti*. As a consequence, there were mycotoxins present in the egg whites. The production of mycotoxins in the egg content may indicate that microscopic fungi competed with other microorganisms. The results showed that the conditions of the henhouse environment significantly influenced the initial eggshell contamination. These main sources of contamination should be taken into consideration when preparing strategies preventing the occurrence of toxin-producing fungal species in table eggs.

## 4. Materials and Methods

### 4.1. Egg Collection

The research was conducted on 240 eggs (class M, average weight 53 g) acquired from hens kept in the cage farming production system (Hy-Line White), deep litter indoor housing system (Hy-Line), free-range production system (Green-Legged Hens) and organic production system (Green-Legged Hens) with the ‘PL-Eko’ certificate. The henhouses were located in central Poland.

### 4.2. Mycobiota Isolation, Media and Culture Conditions

On being laid, half of all egg samples were subjected to surface sterilisation in an aqueous ethanol (70%) solution for 30 seconds. All the eggs were stored at high humidity (95%) and temperature (20–25 °C) in a climate chamber (Binder KBF 115, Tuttlingen, Germany). After two weeks of storage the eggs were broken and divided into fractions. Shredded eggshells were aseptically cultured on Potato Dextrose Agar (PDA, Oxoid, Basingstoke, UK). Eggshell mycobiota were incubated at 20–25 °C for 5–7 days. The purification of cultures was conducted according to the manual [34]. Pure fungal cultures were grown on the PDA medium until the genomic DNA extraction.

### 4.3. DNA Extraction

Forty-one fungal cultures were isolated and purified from eggshells. The following molecular techniques were used in the pre-validated procedure. Mycelia of individual isolates were harvested from the PDA plates after 7 days of incubation. Genomic DNA was extracted using the hexadecyltrimethylammonium bromide (CTAB) method used previously [35]. DNA extracts were stored at −20 °C.

### 4.4. Molecular Species Identification

The species were identified by analysing the sequences of a variable fragment of the translation elongation factor 1α gene (tef-1α). Diagnostic fragments were PCR-amplified using the following primer pair: Ef728M—5′-CATCGAGAAGTTCGAGAAGG-3′ and Tef1R—5′-GCCATCCTTGGAGATACCAGC-3′ [36,37]. Identification of other fungi species was confirmed on the basis of the sequence analysis of the Internal Transcribed Spacers of the ribosomal DNA region (ITS1-ITS2). For the PCR, specific primers were used: ITS4—5’-TCCTCCGCTTATTGATATGC-3′ and ITS5—5’-GGAAGTAAAAGTCGTAACAAGG-3′ [38]. PCRs were conducted in 20 μL volumes using C-1000 thermal cyclers (Bio-Rad, Hercules, CA, USA). Each reaction tube contained 0.4 μL of Phire II HotStart Taq DNA polymerase (Thermo Scientific, Espoo, Finland), 4 μL of 5× PCR buffer, 12.5 pmol of forward/reverse primers, 2.5 mM of each dNTP and 20 ng of fungal DNA. The PCR conditions were as follows: 30 s at 98 °C; 35 cycles of 5 s at 98 °C, 5 s at 63 °C (for tef-1α) or 5 s at 56 °C (for ITS1-ITS2) and 15 s at 72 °C and, finally, 1 min at 72 °C. The amplicons were electrophoresed in 1.5% (*w*/*v*) agarose gels (Invitrogen, Carlsbad, CA, USA) with Midori Green dye (Nippon Genetics Europe GmbH, Dueren, Germany).

For sequence analysis, PCR-amplified DNA fragments were purified with exonuclease I (Thermo Scientific, Espoo, Finland) and shrimp alkaline phosphatase (Thermo Scientific, Espoo, Finland) using the following programme: 30 min at 37 °C and 15 min at 80 °C. DNA fragments were labelled using forward primer and the BigDyeTerminator 3.1 kit (Applied Biosystems, Foster City, CA, USA), according to the method described in Stępień et al. [39] and precipitated with 96% ethanol. Sequences were read using Applied Biosystems equipment (Applied Biosystems, Foster City, CA, USA). The sequences were aligned using BLASTn algorithm to the sequences of reference strains belonging to individual fungal species, deposited in the GenBank Database (National Center for Biotechnology Information, Bethesda, MD, USA). They were assigned to the reference species, where both sequence coverages and nucleotide identities matched the query at 99–100%.

### 4.5. Analysis of Mycotoxins in Egg White

Lyophilised egg white was analysed for the content of selected mycotoxins. The concentrations of the following mycotoxins were measured: type A trichothecenes, that is, scirpentriol (STO), diacetoxyscirpenol (DAS), HT-2 toxin, T-2 toxin, T-2 tetraol, T-2 triol and type B trichothecenes, that is, deoxynivalenol (DON), 3-acetyl-deoxynivalenol (3-AcDON), 15-acetyl-deoxynivalenol (15-AcDON), nivalenol (NIV), fusarenone X (FUS-X). Samples (10 g) were extracted with 100 ml mixture of acetonitrile/water (82:18). The extracts were purified by means of a solid phase extraction, using columns filled with a mixture of active carbon (Draco G 60, 100 mesh), celite (Celite 545) and neutral aluminium oxide (70–230 mesh) at a weight ratio of 1:1:1. Type B trichothecenes were analysed as trimethylsilyl derivatives, using an external model. They were separated chromatographically and analysed individually by means of a gas chromatograph (Hewlett Packard 6890, Waldbronn, Germany) coupled with a mass detector (Hewlett Packard 5972 A, Waldbronn, Germany). Selected ions (SIM) were analysed in type B trichothecenes: for DON—ions 103 and 512; for 3-AcDON—ions 117 and 482; for 15-AcDON—ions 193 and 482; for FUS-X—ions 103 and 570; for NIV—ions 191 and 600. A full-range scan of masses was analysed (100–700 amu) to confirm that analysed toxins were present in the samples. The mass spectrum was compared with the analogical spectrum referring to the standard. Apart from the qualitative analysis, the toxin concentrations were measured. The results were processed with the ChemStation program. Type A trichothecenes were analysed as trifluoroacetyl derivatives. The analysis consisted in searching for selected ions (SIM). The following ions were identified in type B trichothecenes: for STO—ions 456 and 555; for T-2 tetraol—ions 455 and 568; for T-2 triol—ions 455 and 569; for DAS—ions 402 and 374; for HT-2—ions 455 and 327; for T-2—ions 327 and 401. The method of analysis resulted in the following percentages of toxin recovery: STO 82 ± 5.3%; T-2 triol 79 ± 5.1%; T-2 86 ± 3.8%; T-2 tetraol 88 ± 4.0%; FUS-X 79 ± 3.1%; HT-2 91 ± 3.3%; DON 84 ± 3.8%; 3-AcDON 78 ± 4.8%; 15-AcDON 74 ± 2.2% and NIV 81 ± 3.8%. The limit of detection was 0.001 mg/kg.

## Figures and Tables

**Table 1:** Mycobiota strains isolated from the eggs in the present study.

Hen Housing System	Sample Treatment	Identified Fungal Isolates
Cage	Native mycobiota	*Alternaria brassicae* *Alternaria obovoidea* *Botryotrichum spirotrichum* *Chaetomium murorum*
	Surface-sterilised	*Alternaria arborescens* *Alternaria burnsii* *Alternaria tenuissima* *Engyodontium album*
Deep litter housing	Native mycobiota	*Alternaria alternata* *Alternaria tenuissima* *Chaetomium globosum* *Engyodontium album* *Fusarium culmorum* *Fusarium equiseti*
	Surface-sterilised	*Acrostalagmus luteoalbus* *Alternaria arborescens* *Alternaria brassicae* *Alternaria infectoria* *Alternaria multiformis* *Alternaria tenuissima* *Chaetomium globosum* *Penicillium chrysogenum* *Penicillium riseofulvum* *Scopulariopsis brevicaulis* *Trichothecium roseum*
Free range	Native mycobiota	*Alternaria infectoria* *Alternaria tenuissima* *Chaetomium funicola* *Chaetomium globosum* *Epicoccum nigrum* *Fusarium culmorum* *Fusarium tricinctum* *Trichothecium roseum*
	Surface-sterilised	*Alternaria* sp. *Acrostalagmus luteoalbus* *Chaetomium globosum* *Purpureocillium lilacinum*
Organic	Native mycobiota	*Alternaria alternata* *Epicoccum nigrum* *Trichothecium roseum*
	Surface-sterilised	*Alternaria infectoria* *Alternaria multiformis* *Alternaria brassicae* *Alternaria tenuissima* *Chaetomium elatum* *Chaetomium globosum* *Epicoccum nigrum* *Purpureocillium lilacinum* *Trichothecium roseum*

**Table 2:** Mean values and standard deviations of type-A trichothecenes present in egg samples contaminated with *Fusarium* species.

*Fusarium* sp.	Concentration of Type-A Trichothecenes (μg/kg)					
	Scirpentriol	T-2 Tetraol	T-2 Triol	DAS	HT-2	T-2
*Fc*	3 ± 1	5 ± 1	2 ± 1	11 ± 9	16 ± 8	22 ± 7
*Fe*	<LOD	<LOD	<LOD	<LOD	<LOD	<LOD
*Ft*	<LOD	<LOD	<LOD	<LOD	<LOD	<LOD
none	10 ± 9	<LOD	<LOD	<LOD	<LOD	13 ± 9

*Fc—Fusarium culmorum, Fe—F. equiseti, Ft—F. tricinctum*.

**Table 3:** Mean values and standard deviations of type-B trichothecenes present in egg samples contaminated with *Fusarium* species.

*Fusarium* sp.	Concentration of Type-B Trichothecenes (μg/kg) ± SD				
	DON	FUS-X	3-AcDON	15-AcDON	NIV
*Fc*	16 ± 3	2 ± 1	4 ± 2	3 ± 1	55 ± 11
*Fe*	<LOD	<LOD	<LOD	<LOD	<LOD
*Ft*	<LOD	<LOD	<LOD	<LOD	<LOD
none	10 ± 4	<LOD	<LOD	<LOD	19 ± 9

*Fc—Fusarium culmorum, Fe—F. equiseti, Ft—F. tricinctum*.

**Table 4:** The range and mean concentrations of total toxins in eggs laid by hens kept in different housing systems.

Egg Content		Total Toxin Concentrations (μg/kg)	
		Range	Mean
Native mycobiota	Organic	<LOD	-
	Free range	0.1–55	36 ^a^
	Deep litter	0.1–38	27 ^a^
	Cage	<LOD	-
Surface-sterilised	Organic	<LOD	-
	Free range	<LOD	-
	Deep litter	13–19	14
	Cage	<LOD	-

^a^—no differences at the level of significance α = 0.05.

## References

[1] (2006). Commission Regulation (EC) 1881/2006. Off. J. Eur. Commun..

[2] De Reu K., Grijspeerdt W., Messensa M., Heyndrickxa M., Uyttendaeleb J., Debevereb L., Hermana L. (2006). Eggshell factors influencing eggshell penetration and whole egg contamination by different bacteria, including Salmonella enteritidis. Int. J. Food Microbiol..

[3] Gantois I., Ducatelle R., Pasmans F., Haesebrouck F., Gast R., Humphrey T.J., Van Immerseel F. (2009). Mechanisms of egg contamination by *Salmonella enteritidis*. FEMS Microbiol. Lett..

[4] Corry J.E.L., Mead Red G.C. (2007). Microbiological analysis of eggs and egg products. Microbiological Analysis of Red Meat, Poultry and Eggs.

[5] Gros R.V., Nichita I., Șereș M., Ilie M.S., Marcu A., Cucerzan A., Tîrziu E. (2015). Study of the fungi dynamics in a poultry house with permanent litter. Lucr. St. Med. Vet..

[6] Harry E.G., Cooper D.M. (1970). The treatment of hatching eggs for the control of egg transmitted aspergillosis. Br. Poult. Sci..

[7] Girma G., Abebaw M., Zemene M., Mamuye Y., Getaneh G. (2016). A Review on Aspergillosis in Poultry. J. Vet. Sci. Technol..

[8] Szablewski T., Stuper K., Cegielska-Radziejewska R., Kijowski J., Perkowski J. (2010). Ergosterol as an indicator of the presence of microscopic fungi in eggs for human consumption produced in different husbandry systems. Poult. Sci..

[9] Rohweder D., Valenta H., Sondermann S., Schollenberger M., Drochner W., Pahlow G., Döll S., Dänicke S. (2011). Effect of different storage conditions on the mycotoxin contamination of *Fusarium culmorum*-infected and non-infected wheat straw. Mycotoxin Res..

[10] Kokkonen M., Ojala L., Parikka P., Jestoi M. (2010). Mycotoxin production of selected *Fusarium* species at different culture conditions. Int. J. Food Microbiol..

[11] Stuper-Szablewska K., Szablewski T., Cegielska-Radziejewska R., Ostrowska A., Matysiak A., Perkowski J. (2014). Contamination of different kind of litter by microscopic fungi from hen house. Apar. Bad. Dydakt..

[12] Skóra J., Gutarowska B., Pielech-Przybylska K., Stępień Ł., Pietrzak K., Piotrowska M., Pietrowski P. (2015). Assessment of microbiological contamination in the work environments of museums, archives and libraries. Aerobiologia.

[13] Sypecka Z., Kelly M., Brereton P. (2004). Deoxynivalenol and Zearalenone Residues in Eggs of Laying Hens Fed with a Naturally Contaminated Diet, Effects on Egg Production and Estimation of Transmission Rates from Feed to Eggs. J. Agric. Food Chem..

[14] Binder E.M., Tan L.M., Chin L.J., Handl J., Richard J. (2007). Worldwide occurrence of mycotoxins in commodities, feeds and feed ingredients. Anim. Feed Sci. Technol..

[15] Desjardins A.E. (2006). Fusarium Mycotoxins Chemistry, Genetics and Biology.

[16] Zur G., Shimoni E., Hallerman E., Kashi Y. (2002). Detection of *Alternaria* fungal contamination in cereal grains by a polymerase chain reaction-based assay. J. Food Prot..

[17] Nielsen K.F., Gravesen S., Nielsen P.A., Andersen B., Thrane U., Frisvad J.C. (1999). Production of mycotoxins on artificially and naturally infested building materials. Mycopathologia.

[18] Fogle M.R., Douglas D.R., Jumper C.A., Straus D.C. (2007). Growth and mycotoxin production by *Chaetomium globosum*. Mycopathologia.

[19] Provost N., Shi C., She Y., Cyr T., Miller D. (2013). Characterization of an antigenic chitosanase from the cellulolytic fungus *Chaetomium globosum*. Med. Mycol..

[20] Piskorska-Pliszczynska J., Mikolajczyk M., Warenik-Bany M., Maszewski S., Strucinski P. (2014). Soil as a source of dioxin contamination in eggs from free-range hens on a Polish farm. Sci. Total Environ..

[21] Macêdo D.P.C., Neves R.P., De Souza-Motta C.M., Magalhães O.M.C. (2007). *Engyodontium album* fungaemia, the first reported case. Braz. J. Microbiol..

[22] Holt P.S., Davies R.H., Dewulf J., Gast R.K., Huwe J.K., Jones D.R., Waltman D., Willian K.R. (2011). The impact of different housing systems on egg safety and quality. Poult. Sci..

[23] Wall H., Tauson R., Sorgjerd S. (2008). Bacterial contamination of eggshells in furnished and conventional cages. J. Appl. Poult. Res..

[24] Scherm B., Balmas V., Spanu F., Pani G., Delogu G., Pasquali M., Migheli Q. (2013). *Fusarium culmorum*, causal agent of foot and root rot and head blight on wheat. Mol. Plant Pathol..

[25] Kuzdraliński A., Nowak M., Szczerba H., Dudziak K., Muszyńska M., Leśniowska-Nowak J. (2017). The composition of *Fusarium* species in wheat husks and grains in south-eastern Poland. J. Integr. Agric..

[26] Stępień Ł., Waśkiewicz A., Urbaniak M. (2016). Wildly growing asparagus (*Asparagus officinalis* L.) hosts pathogenic Fusarium species and accumulates their mycotoxins. Microb. Ecol..

[27] Ciegler A. (1978). Fungi that produce mycotoxins, Conditions and occurrence. Mycopathologia.

[28] Stępień Ł., Gromadzka K., Chełkowski J. (2012). Polymorphism of mycotoxin biosynthetic genes among *Fusarium* equiseti isolates from Italy and Poland. J. Appl. Genet..

[29] Prelusky D.B., Hamilton R.M.G., Trenholm H.L. (1989). Transmission of Residues to Eggs Following Long-Term Administration of 14C-Labelled Deoxynivalenol to Laying Hens. Poult. Sci..

[30] Prelusky D.B., Trenholm H.L., Hamilton R.M.G., Miller J.D. (1987). Transmission of [14C] Deoxynivalenol to Eggs following Oral Administration to Laying Hens. J. Agric. Food Chem..

[31] Valenta H., Dänicke S. (2005). Carry-over of deoxynivalenol into eggs of laying hens—Preliminary results. Mycotoxin Res..

[32] Tangni E.K., Waegeneersa N., Van Overmeireb I., Goeyensb L., Pussemier L. (2009). Mycotoxin analyses in some home produced eggs in Belgium reveal small contribution to the total daily intake. Sci. Total Environ..

[33] Escrivá L., Font G., Manyes L., Berrada H. (2017). Studies on the Presence of Mycotoxins in Biological Samples: An Overview. Toxins.

[34] Leslie J.F., Summerell B.A. (2006). The Fusarium Laboratory Manual.

[35] Stępień Ł., Jestoi M., Chełkowski J. (2013). Cyclic hexadepsipeptides in wheat field samples and esyn1 gene divergence among enniatin producing *Fusarium avenaceum* strains. World Mycotoxin J..

[36] Carbone I., Kohn L.M. (1999). A method for designing primer sets for speciation studies in filamentous ascomycetes. Mycologia.

[37] Kullnig-Gradinger C.M., Szakacs G., Kubicek C.P. (2002). Phylogeny and evolution of genus *Trichoderma* a multigene approach. Mycol. Res..

[38] White T.J., Bruns T., Lee S., Taylor J., Innis M.A., Gelfand D.H., Shinsky J.J., White T.J. (1990). Amplification and direct sequencing of fungal ribosomal RNA genes for phylogenetic. PCR Protocols, a Guide to Methods and Applications.

[39] Stępień Ł., Waśkiewicz A. (2013). Sequence divergence of the enniatin synthase gene in relation to production of beauvericin and enniatins in *Fusarium* species. Toxins.

